# Response to “Body temperature correlates with mortality in COVID-19 patients”

**DOI:** 10.1186/s13054-020-03186-w

**Published:** 2020-07-24

**Authors:** Anne M. Drewry, Richard Hotchkiss, Erik Kulstad

**Affiliations:** 1 Surgical Intensive Care Unit, Critical Care Medicine, Department of Anesthesiology, Washington University School of Medicine, 660 S. Euclid Ave., Campus Box 8054, St. Louis, MO 63110 USA; 2 Anesthesiology, Medicine, Surgery, and Developmental Biology, Washington University School of Medicine, 660 S. Euclid Ave., Campus, Box 8054, St. Louis, MO 63110 USA; 3 Department of Emergency Medicine, UT Southwestern Medical Center, 5323 Harry Hines Blvd., Dallas, TX 75390 USA

Dear Editor:

Tharakan et al. found a trend of increased mortality with poor temperature control in severely ill COVID-19 patients and suggest further studies to determine if controlling high temperature might alleviate the inflammatory response and improve outcomes [1]. We agree that temperature is an important factor in critical illness, but worry that the lack of adjustment made in the analysis of the authors’ data may provide spurious associations, and that in fact, elevated temperatures may actually be of benefit.

Multiple aspects of both humoral and cellular immunity (including antibody production, T lymphocyte trafficking, T cell adhesion and migration, heat shock protein 90 (Hsp90)-induced α4 integrin activation and signaling, and macrophage function) are boosted by elevated temperature, and numerous studies have found no benefit to treating fever of infectious origin [2]. After adjusting for identified confounders in a sepsis subgroup of mechanically ventilated patients, a maximum temperature ≥ 39.58 °C was not a predictor of death, and a maximum temperature between 38.3 °C and 39.48 °C was associated with survival [3]. A prospective study found that afebrile patients have higher 28-day mortality (37.5% vs 18.2%), increased acquisition of secondary infections (35.4% vs. 15.9%), and suppressed HLA-DR expression suggestive of monocyte dysfunction over time [4]. In further investigation of the effects of warming septic patients, a pilot randomized controlled study (ClinicalTrials.gov Identifier: NCT02706275) has recently completed enrollment.

With the results of the Induced Hypothermia in Patients With Septic Shock and Respiratory Failure (CASS) randomized, controlled study, as well as a more recent but smaller randomized study of fever reduction in sepsis showing potential harm [5], we would caution against further pursuing aggressive temperature reduction in patients with infectious etiology for fever. On the other hand, the potential for warming appears promising, with studies in various stages of progress (NCT04426344).

## Authors’ response

Serena Tharakan and Kiyotake Ishikawa

We thank Drewry et al. for their interest in our Research Letter. As the authors pointed out, we agree and noted in our letter that we were limited by our inability to adjust for disease severity due to the lack of relevant parameters in our dataset. Still, the correlation between maximum body temperature and mortality from COVID-19 was obvious and showed a clear trend. Thus, we believe our conclusion remains solid: maximum body temperature during the course of COVID-19 is a good and easily obtainable predictor of worse outcomes.

Drewry et al. discussed the potential benefit of elevated body temperature based on the results of clinical trials that primarily included patients with bacterial sepsis. However, there is a lack of evidence to suggest that sepsis due to SARS-CoV-2 and bacterial sepsis have the same pathophysiology or can be treated in the same way. There are notable differences between sepsis due to virus and sepsis due to bacteria [6]. Remy et al. [7] state that the therapeutic approach to sepsis due to SARS-CoV-2 should be differentiated from that of bacterial sepsis, due to temporal differences in the production of cytokines such as IL-6.

Elevated body temperatures may augment the immune response to SARS-CoV-2 and potentially inhibit viral replication. However, it is important to consider this argument in the context of the widespread damage that high body temperature might cause on top of virally induced systemic inflammation. Fever increases metabolic demand and oxygen consumption of many organs, which may exacerbate tissue injury caused by systemic inflammation. For example, it is known that COVID-19 is associated with increased risk of myocardial injury [8]. Fever can also negatively impact cardiac function and has been shown to aggravate necrosis and no-reflow during myocardial infarction in animal models [9]. The acute respiratory distress and respiratory failure associated with COVID-19 is likely also worsened by fever and might be improved by controlling body temperature. Manthous et al. showed that in critically ill febrile patients, reducing body temperature from 39 to 37 °C lowered oxygen consumption, unloaded the cardiopulmonary system, and facilitated resuscitation of patients with hypoxemic respiratory failure or limited oxygen delivery [10].

Given the observed high mortality in patients with elevated temperatures and the paucity of data on temperature management for COVID-19, it is important to explore every potential approach to identify optimal temperature management strategies. This includes warming studies as Drewry et al. mention, as well as maintaining normothermia and hypothermia.

## Data Availability

N/A
